# Role and mechanisms of callose priming in mycorrhiza-induced resistance

**DOI:** 10.1093/jxb/eraa030

**Published:** 2020-01-27

**Authors:** Neus Sanmartín, Victoria Pastor, Julia Pastor-Fernández, Victor Flors, Maria Jose Pozo, Paloma Sánchez-Bel

**Affiliations:** 1 Metabolic Integration and Cell Signaling Laboratory, Plant Physiology Section, Unidad Asociada al Consejo Superior de Investigaciones Científicas (EEZ-CSIC)-Department of Ciencias Agrarias y del Medio Natural, Universitat Jaume I, Castellón, Spain; 2 Department of Soil Microbiology and Symbiotic Systems, Estación Experimental del Zaidín (CSIC), Granada, Spain; 3 Lancaster University, UK

**Keywords:** *Botrytis cinerea*, callose, mycorrhiza-induced resistance, priming, starch degradation, sugar transport

## Abstract

Mycorrhizal plants display enhanced resistance to several pathogens. However, the molecular mechanisms regulating mycorrhiza-induced resistance (MIR) are still elusive. We aim to study the mechanisms underlying MIR against *Botrytis cinerea* and the role of callose accumulation during this process. Mycorrhizal tomato plants inoculated with *Rhizoglomus irregularis* displayed callose priming upon *B. cinerea* infection. The callose inhibitor 2-deoxy-d-glucose abolished MIR, confirming the relevance of callose in the bioprotection phenomena. While studying the mechanisms underlying mycorrhiza-induced callose priming, we found that mycorrhizal plants display an enhanced starch degradation rate that is correlated with increased levels of *β-amylase1* transcripts following pathogen infection. Starch mobilization in mycorrhizal plants seems coordinated with the increased transcription of sugar transporter and invertase genes. Moreover, the expression levels of genes encoding the vesicular trafficking proteins ATL31 and SYP121 and callose synthase PMR4 were higher in the mycorrhizal plants and further boosted by subsequent pathogen infection. All these proteins play a key role in the priming of callose accumulation in Arabidopsis, suggesting that callose priming is an induced resistance mechanism conserved in different plant species. This evidence highlights the importance of sugar mobilization and vesicular trafficking in the priming of callose as a defence mechanism in mycorrhiza-induced resistance.

## Teaser

We prove the relevance of callose priming in mycorrhiza-induced resistance against shoot pathogens and studied the main mechanisms regulating it such as starch mobilization, sugar delivery, and vesicular trafficking.

## Introduction

Beneficial microorganism–plant interactions are widespread in nature. Among such interactions, >80% of plants are associated with arbuscular mycorrhiza fungi (AMF), which are soil-borne obligate biotrophs belonging to the Glomeromycota phylum. AMF are found in practically all agricultural and natural environments. These fungi establish a mutualistic symbiosis with plant roots, forming specialized intracellular structures in the root cortex known as arbuscules, where the interchange of nutrients between the symbionts occurs (Gutjahr and Parniske, 2013). Once symbiosis is well established, the plant provides photosynthates and lipids to the fungus and, in return, the AMF improve the plant mineral nutrients and water uptake. In addition to these benefits, AM plants present enhanced tolerance to abiotic and biotic stresses (Miransari, 2010; Jung *et al.*, 2012; Sánchez-Bel et al., 2016; Rivero *et al.*, 2018).

Several studies have shown that AM plants are more resistant to soil-borne and shoot pathogens. Upon biotic challenges, AM plants mount defences in a faster and more efficient manner, representing a phenomenon known as defence priming (Pozo and Azcón-Aguilar, 2007; Jung *et al.*, 2012; Martinez-Medina *et al.*, 2016; Mauch-Mani *et al.*, 2017). Hence, priming is suggested as the mechanism underlying mycorrhiza-induced resistance (MIR; Jung *et al.*, 2012; Balmer *et al.*, 2015). Earlier studies have revealed systemic protection by mycorrhiza against root pathogens associated with the enhanced accumulation of pathogenesis-related (PR) proteins, phenolic compounds, and callose-containing cell wall appositions at pathogen entrance points (Cordier *et al.*, 1998). *Funneliformis mosseae* in symbiosis with tomato plants alleviated *Alternaria solani* disease by priming some defence-related genes, and the authors showed that oxylipin [jasmonic acid (JA)]-dependent defences support MIR since JA-deficient *spr2* mutant plants did not display the induced resistance (Song *et al.*, 2015). Oxylipin-related responses have also been reported in *Rhizoglomus irregulairs-*induced resistance to *Botrytis cinerea* (Sánchez-Bel et al., 2016).

The benefits of symbiosis have also been reported in AM plants facing abiotic and biotic stresses simultaneously. Non-mycorrhizal (NM) plants usually react to nutritional stress by preparing their metabolism to address the nutrient deficiency at the cost of biotic stress defences. However, AM plants buffer the growth–defence balance by maintaining functional biotic defences under nitrogen starvation (Sánchez-Bel *et al.*, 2018).

One of the most intriguing cellular defence responses against pathogens is the deposition of β-glucan polysaccharide callose. This sugar polymer reinforces plant cell walls against attackers, blocks their entrance, and provides the plant with additional time to activate subsequent defence mechanisms if needed. AM plants infected with *Blumeria graminis* show increased papillae formation at penetration sites (Mustafa *et al.*, 2017). Moreover, AMF can trigger callose accumulation in wheat following chitosan infiltration (Pérez-de-Luque *et al.*, 2017), although the underlying molecular mechanisms have not been explored.

Callose synthase is the enzyme responsible for the accumulation of callose at the papillae. In Arabidopsis plants, 12 callose synthase genes named *GSL1* to *GSL12* have been identified. Of these genes, *GSL5* (also named *PMR4*), is the callose synthase expressed upon pathogenic infection. In tomato plants, two orthologues of Arabidopsis *PMR4* were found, but only one orthologue has been shown to share the same function (Huibers *et al.*, 2013).

Despite the relevance of callose accumulation in induced defences, knowledge regarding the sugar supply required for callose formation is lacking. It has been suggested that indolic glucosinolates in Arabidopsis (Clay *et al.*, 2009) or indolic benzoxazinoids in maize (Ahmad *et al.*, 2011) may provide precursors for callose accumulation. Recently, we showed that starch degradation and subsequent vesicular sugar transport mediate the priming of callose deposition in Arabidopsis (Gamir *et al.*, 2018). The final steps of callose accumulation at the cell wall are regulated by interactions between SNARE proteins. The SNARE complex is required for papilla formation at the fungal entry site. In Arabidopsis, the overexpression of *AtATL31* (*Arabidopsis Toxicos en Levadura 31*) confers resistance to powdery mildew by an enhancement of callose accumulation at early time points of the infection (Maekawa *et al.*, 2014). Recently, our group has shown that in Arabidopsis, *ATL31* and its interactor *SYP121* regulate indole-3-carboxylic acid (I3CA) callose priming downstream of *BAM1* (Gamir *et al.*, 2018).

The modulation of sugar pools in plants is considered to function as a signal to prime immune responses (Gómez-Ariza *et al.*, 2007). Sugar allocation between source leaves and sink organs is of primary importance for both AM symbiosis and plant–pathogen interactions. In addition to plant–pathogen interactions, specific sugars are proposed to function as priming agents in several plant–pathogen systems (Bolouri Moghaddam and Van den Ende, 2012). Although sugar partitioning in plants is a complex topic, exogenous applications of specific sugars, such as galactinol (Kim *et al.*, 2008), trehalose (Singh *et al.*, 2011), and oligogalacturonides, can trigger PR gene expression among other plant immune responses (Bolouri Moghaddam and Van den Ende, 2012). Recently, the concept of ‘sweet immunity’ has been demonstrated in lettuce against *B. cinerea* (Tarkowski *et al.*, 2019). In addition, the availability of carbohydrates affects plant resistance against shoot pathogens (reviewed by Trouvelot *et al.*, 2014). Thus, both plants and pathogens have evolved mechanisms to modulate the carbohydrate content and flux targeting different steps of the carbohydrate biosynthesis, transport, and degradation processes (Essmann *et al.*, 2008; Scharte *et al.*, 2009; Asai *et al.*, 2016). In fact, during plant–pathogen interactions, the expression of genes encoding cell wall invertases is induced by elicitors in different plant species (Proels and Hückelhoven, 2014). Thus, invertases and the released sugars may act as signals for defence against pathogens (Kocal *et al.*, 2008).

The sugar and lipids provided by the host to AMF are among the most relevant benefits obtained by the fungus during symbiosis (Smith and Smith, 2011; Rich *et al.*, 2017). Regarding the potential role of sugars in defence, the extent to which the differential response of AM plants to diseases is due to mycorrhizal-related changes in carbohydrate metabolism remains unexplored. Several sugar transporter genes belonging to the *SUT* family (*SUT1*, *2*, and *4*) and *SWEET* family, some invertase genes (*LIN6*), and sucrose synthases have higher levels during symbiosis (Schaarschmidt *et al.*, 2007; Roth and Paszkowski, 2017). However, the role of sugar mobilization during symbiosis is far more complex; for example, *SUT2* has been suggested to play a sensing role rather than simply playing a role in sugar transport (Barker *et al.*, 2000), although this hypothesis has not been further supported.

In the present study, we prove the relevance of the priming of callose deposition in MIR against the fungal foliar pathogen *B. cinerea*. Moreover, we shed some light on the mechanisms regulating such an enhanced production of callose, which seems to rely on enhanced starch mobilization and sugar delivery to produce such defence structures. We explored the regulation of a stress-related β-amylase and several invertases and sugar transporters that probably mediate callose deposition priming during MIR. Furthermore, we analysed the relevance and contribution of vesicular transport delivering glucose to the cell wall for this defence mechanism.

## Materials and methods

### Plant material and AMF inoculation

Tomato seeds (*Solanum lycopesicum* L. cv. Better Boy) were sterilized with 10% HCl (v/v) and rinsed abundantly with sterile water. Seeds were germinated in sterile vermiculite in a growth chamber with a 16 h light period, 70% relative humidity, and 26 °C during the day and 18 °C during the night. Later, seedlings were transplanted to 200 cm^3^ pots with sterile vermiculite. The AMF *Rhizoglomus irregularis* (BEG 121) (formerly *Glomus intraradices*), which was maintained as a soil–sand-based inoculum, was added into the pots at 15% (v/v). For control plants, a 15% (v/v) mixture of soil and sand without AMF inoculum was added. To homogenize the microbial populations, control plants were irrigated with a filtrate (20 µm) of the AMF inoculum. Photoperiod and temperature were maintained in the growth chamber. Twice a week, tomato plants were watered with Long Ashton solution (Hewitt, 1996) with 25% of the standard phosphorus concentration. Plants grew for an additional 4 weeks before pathogen infection to ensure establishment of symbiosis.

### *Botrytis cinerea* infection

*Botrytis cinerea* CECT2100 (Spanish collection of type cultures, Universidad de Valencia, Burjassot, Spain) was grown from 2 weeks in PDA (potato dextrose agar) plates supplemented with tomato leaves (40 mg ml^–1^). Conidia were collected and pre-germinated in Gambor’s B5 medium (Duchefa, Haarlem, The Netherlands) supplemented with 10 mM sucrose and 10 mM KH_2_PO_4_ for 2 h in the dark without shaking. Plant infection was performed on intact plants at 100% relative humidity as described by Vicedo *et al.* (2009). Plants where inoculated by spraying the third and fourth leaf with a 10^6^ ml^−1^*B. cinerea* spore suspension.

At 72 hours post-infection (hpi), leaves were collected at −80 °C to assess the gene expression and sugar levels, and some leaves were collected and kept in ethanol to study the callose deposition.

*Botrytis cinerea* infection and sample collection were carried out during the diurnal part of the day, 3 h after the turning on of the lights in the phytotron.

### Brefeldin A and 2-deoxy-d-glucose treatment

Detached leaves of NM and AM plants were used to study the effects of treatments with the vesicular trafficking inhibitor brefeldin A (BFA) and the callose synthase competitive inhibitor 2-deoxy-d-glucose (2DDG).

BFA treatment was performed as follows: the petioles of the third and fourth detached leaves were immersed in a solution of 100 µg ml^–1^ BFA (Sigma Aldrich) and 5 mM EDTA-Na_2_ (Panreac Química SA). BFA solution was prepared as described by Steele-King *et al.* (1999). For 2DDG treatment and uptake, the petioles of the third and fourth detached leaves were immersed in a solution of 1 mM 2DDG (Sigma Aldrich) and 5 mM EDTA-Na_2_. Control leaves were placed in the same type of tubes with water and 5 mM EDTA-Na_2_. Plants were treated 24 h before the *B. cinerea* infection.

For these experiments, the infection was carried out with 5 µl drops of a 10^6^ ml^−1^*B. cinerea* spore suspension. Four leaves were used in each treatment, with five drops per leaf, one in each leaflet (*n*=20). The experiment was repeated twice, giving similar results. At 72 hpi, leaves were collected and put in ethanol to study the lesion diameter.

### Mycorrhizal colonization levels

Samples of each plant root system were collected to verify the mycorrhizal colonization. Roots were cut into 2 cm segments and stained using the method described by Vierheilig *et al.* (1998), to stain the fungal structures inside roots. To assess the percentage of root colonization by the AMF we used the grid-line intersection method (Giovannetti and Mosse, 1980) in a Nikon Eclipse 50i microscope under bright-field conditions.

### Callose deposition and fungal lesion

Callose deposition was evaluated at 72 hpi by staining the leaves with aniline blue as described by Ton and Mauch-Mani (2004). Stained leaves were photographed under an epifluorescence microscope with a UV filter (Nikon Eclipse 80i). Callose deposition was determined by the quantification of yellow pixels with respect to total pixels of the leaf using GIMP software (GNU Image Manipulation Program, http://gimp.org).

Fungal lesions in BFA and 2DDG treatments were determined as lesion diameter using GIMP software. Leaves were first stained with lactophenol trypan blue (Saha *et al.*, 1988) and then photographed under an epifluorescence microscope with a UV filter (Nikon Eclipse 80i).

### RNA extraction and gene expression analysis

Samples of frozen leaves were ground in liquid nitrogen and stored at −80 °C. RNA was extracted essentially as described (Kiefer *et al.*, 2000) with some changes. In short, 1 ml of Trizol was added to 100 mg of ground fresh leaves. Samples were centrifuged, and the supernatant was collected in a new tube to which 220 µl of CHCl_3_ were added. Next, a second centrifugation was carried out and the supernatant was collected in a new tube. A 350 µl aliquot of isopropanol and 350 µl of 0.8 M citrate/1.2 mM NaCl were added and mixed. After centrifugation, the supernatant was removed, and the pellet was rinsed twice with 70% ethanol. The pellet was dried and dissolved in 25 µl of nuclease-free water.

RNA samples were treated with DNase I (Thermo Scientific) to remove the remaining DNA. Afterwards, 1 μg of RNA was annealed to oligo(dT) and reverse transcribed using a PrimeScript™ RT Reagent Kit (TAKARA) to synthesize the cDNA. Real-time PCR was performed with a Maxima SYBR Green/ROX qPCR Master Mix (2×) (Thermo Scientific) in a StepOne instrument (Applied Biosystems).

To improve the amplification efficiency, serial dilutions of cDNA were performed to create a standard curve. Three technical replicates were done for each sample. The specificity of quantitative reverse transcription–PCR (RT–qPCR) amplification was verified by the presence of a single peak in the melting temperature curve analysis. mRNA levels were quantified using the comparative 2^−Δ(ΔCt)^ method (Livak and Schmittgen, 2001). Tomato elongation factor 1*α* (EF-1*α*) and tomato actin 52 (ACT-52) were used as the housekeeping genes to normalize the expression values. Relative expression of mRNA levels was calculated from the difference in threshold cycle (∆Ct) among the studied genes (*BAM1*, *SUS1*, *SUS3*, *LIN6*, *SUT1*, *SUT2*, *SUT4*, *ATL31*, *SYP121*, *SWEET15*, *SWEET17*, *SWEET4*, and *PMR4*) and housekeeping genes. Primers are detailed in Supplementary Table S1 at *JXB* online.

### DNA extraction and quantification of *Botrytis cinerea* infection

To quantify *B. cinerea* infection, DNA was extracted from infected leaves as described by Edwards *et al.* (1991). Briefly, extraction buffer (200 mM Tris–HCl pH 7.5, 250 mM NaCl, 25 mM EDTA-Na_2_, 0.5% SDS) was added to 50 mg of ground material. Samples were centrifuged, and the supernatant was transferred to a new tube containing 300 µl of isopropanol. Another centrifugation was performed, and the supernatant was discarded. The pellet was dried and dissolved in 50 µl of nuclease-free water. Later, DNA was amplified by quantitative PCR and *B. cinerea* infection was quantified by comparing the levels of the *B. cinerea* tubulin gene *Bc-TUB* versus the levels of the *Sl-EF1α* gene. The primers used for the gene expression analysis and the plant and fungal genomic DNA amplification are detailed in Supplementary Table S1.

### Liquid chromatography coupled to ESI mass spectrometry

#### Targeted hormonal analysis

Hormones were analysed as described as Sánchez-Bel *et al.* (2018). In brief, a pool of internal standards, which contains dehydrojasmonic acid (dhJA) and jasmonic acid-isoleucine-d_6_ (JA-Ile-d_6_), was added to 30 mg of powdered freeze-dried material. Then, external calibration curves of each pure standard (dhJA and JA-Ile-d_6_) were used for the precise JA and JA-Ile quantification, respectively. The extraction was carried out in a mixer mill. After centrifugation, the supernatant was placed in a new tube, and the pH was adjusted with acetic acid to 2.5–2.7. Diethyl ether was added to create two phases, and a centrifugal evaporator (Speedvac) was used to concentrate both organic fractions until dryness. A 1 ml aliquot of MeOH/H_2_O with 0.01% HCOOH (10:90) was added, to a final concentration of internal standards of 100 ng ml^−1^. The targeted hormonal analysis was performed in an Acquity ultraperformance liquid chromatography system (UPLC; Waters, Mildford, MA, USA) coupled to a triple quadrupole mass spectrometer (TQD, Waters, Manchester, UK). The conditions used were described by Sánchez-Bel et al. (2016).

#### LC-ESI full-scan mass spectrometry for sugar analysis

A 300 mg aliquot of freeze-dried material was powdered and 1 ml of MeOH 10% was added. After centrifugation, the supernatant was collected and filtered with a 0.2 µm filter (Regenerated Cellulose Filter, 0.20 μm, 13 mm D. pk/100; 263 Teknokroma). Samples were kept at −20 °C for further analysis.

Untargeted LC-ESI full-scan MS was done as detailed by Gamir *et al.* (2014). Briefly, 20 μl of the filtered supernatant were injected in an Acquity UPLC system (Waters, www.waters.com) interfaced with a hybrid quadrupole time-of-flight mass spectrometer (QTOF Premier). The identification of the detected signals, as described by Sánchez-Bel *et al.* (2018), was carried out by introducing a second fragmentation function into the TOF analyser. To obtain the fragmentation of each analyte, a new function was programmed in a t-wave ranging from 5 eV to 45 eV. Analytes were eluted using a gradient of MeOH and H_2_O with 0.01% HCOOH.

Sugars were identified by comparing their fragmentation spectrum in the Massbank (https://massbank.eu) or the Metlin databases (https://metlin.scripps.edu).

#### Bioinformatic processing of metabolomic signals

The Databridge tool was used to transform data from the .raw format, obtained with Masslynx 4.1 software (Masslynx 4.1, Waters), to .cdf files. Then, signals were processed using the software R (http://www.r-project.org/). The XCMS algorithm (www.bioconductor.org; Smith *et al.*, 2006) was used to obtain the peak peaking, grouping, and signal corrections. Metabolite amounts were analysed based on the normalized peak area units relative to the dry weight. A non-parametric Kruskal–Wallis test (*P*<0.05) was performed to test the differences among treatments.

### Statistical analysis

Statgraphics-plus software for Windows V.5 (Statistical Graphics Corp., MD, USA) was used to determine the statistical analysis by ANOVA, using Fisher’s least significant difference (LSD) at 99.5% to compare treatments. Means are shown with their SE. Three pooled plants were sampled for each experiment, and each experiment was repeated a minimum of three times.

## Results

### Priming of callose deposition plays an important role in mycorrhiza-induced resistance against *B. cinerea*

**Fig. 1.**
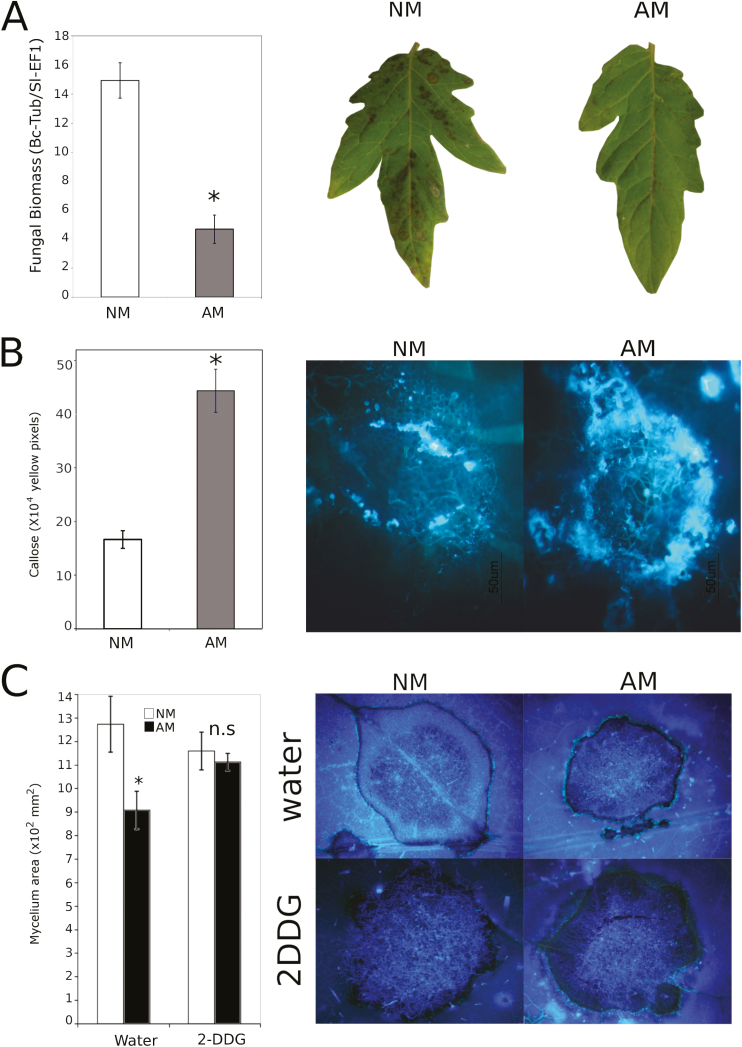
Mycorrhiza-induced resistance (MIR) and the role of callose deposition priming in MIR. (A) Level of infection with 10^6^ spores ml^–1^ of *Botrytis cinerea* at 72 hpi in non-mycorrhizal (NM) and mycorrhizal (AM) plants measured as the ratio of expression of the *Tubulin* gene (*B. cinerea*) relative to *EF1α* (*S. lycopersicum*). (B) Callose levels in NM and AM plants at 72 hpi. (C) Mycelium diameter after drop inoculation of *B. cinerea* 72 hpi in NM (white bars) and AM (black bars) plants treated 24 h before infection with 2DDG (2-deoxy-d-glucose) to inhibit callose deposition or treated with water as a control. The experiment was repeated three times with the same results. The error bars represent the SE of the mean, and the statistically significant differences are represented by (*) (*t*-test; n=18). (This figure is available in colour at *JXB* online.)

Here, we confirmed that MIR was functional in tomato plants against *B. cinerea* as previously reported (Sánchez-Bel *et al*., 2016). Compared with NM plants, the inoculation of tomato plants with the AMF *R. irregularis* led to a significant reduction in the disease symptoms at 72 hpi (Fig. 1A). The disease incidence was assessed by quantifying the fungal biomass through qPCR measuring the presence of the fungal gene *Bc-TUB* versus the plant gene *Sl-EF1α*. The fungal biomass in the AM plants was reduced by 66% compared with that in the NM plants.

A common plant response to pathogen penetration is the formation of papillae surrounding the infection site, which includes callose. To determine whether MIR against *B. cinerea* was mediated by enhanced callose deposition, the control and AM plants were infected with the fungus, and callose accumulation was quantified. Compared with the NM plants, the AM tomato plants showed a priming in callose deposition (Fig. 1B). To investigate whether this callose accumulation is an important component in MIR, the plants were infiltrated with 2-DDG, which is a competitive inhibitor of callose synthase, 24 h prior to inoculation with *B. cinerea*. The infiltration resulted in almost complete inhibition of callose accumulation and the impairment of MIR (Fig. 1C), whereas in the water-infiltrated plants, MIR against *B. cinerea* was fully functional. Since it has been reported that transient nitrogen starvation of AM tomato plants also results in a partial impairment of MIR against *B. cinerea* (Sánchez-Bel et al., 2016), we measured the callose deposition rate in NM and AM plants infected with *B. cinerea* with or without exposure to 48 h of nitrogen starvation prior to the infection (Supplementary Fig. S1). The results showed that under nitrogen starvation, the callose deposition in the NM and AM plants was almost abolished, and, accordingly, these plants were unable to prevent the entrance of the pathogen, resulting in enhanced susceptibility.

Hormonal regulation is known to be among the main plant elements responsive to pathogens. Mycorrhizal symbiosis strongly alters the hormone homeostasis of the plant, and this alteration has been suggested to be involved in MIR against *B. cinerea* (Sánchez-Bel et al., 2016). Notably, several previous studies suggested that some oxylipins play a regulatory role in callose accumulation, since JA and other oxylipins are thought to regulate the glucan synthase complex in Arabidopsis (Ton and Mauch-Mani, 2004; Flors *et al.*, 2008; García-Andrade *et al.*, 2011). The main defence-related hormones were studied to explore the potential link between altered hormone levels and callose priming. The quantification of the hormones in the oxylipin pathway revealed that the production of all hormones was induced following *B. cinerea* infection regardless of the mycorrhizal status of the plant. JA displayed a priming profile that was significantly higher in the AM plants upon infection than that in the infected NM plants. However, the accumulation of the hormone conjugate JA-Ile was enhanced upon *B. cinerea* infection, although the levels in the infected AM plants remained lower than that in the infected NM plants (Supplementary Fig. S2).

### Starch mobilization probably fuels the priming of callose deposition

**Fig. 2.**
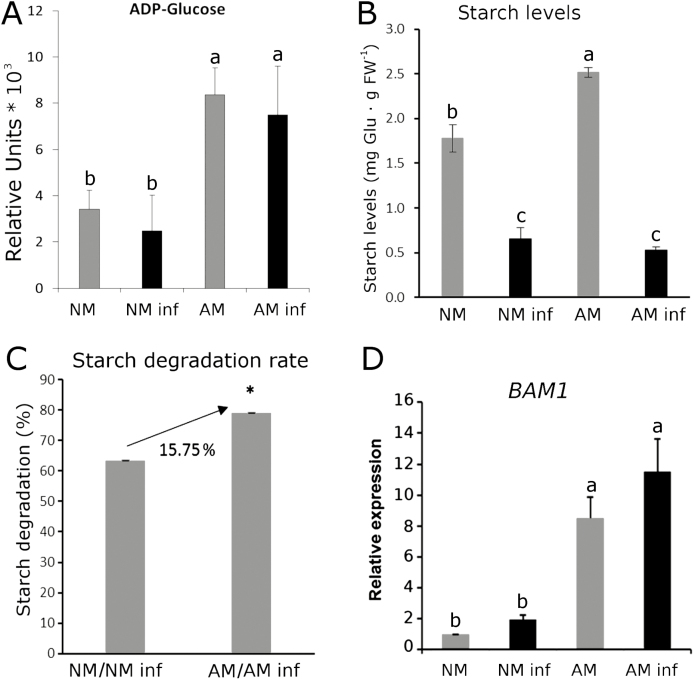
Starch metabolism in the mycorrhizal (AM) plants was higher than that in non-mycorrhizal (NM) plants regardless of the pathogenic infection. (A) Content of the main starch precursor ADP-glucose, (B) starch content, (C) starch degradation represented as the difference between the starch levels before and after the challenge, and (D) relative gene expression of β*-amylase 1* (*BAM1*) in the non-infected (grey bars) and infected (black bars) NM and AM plants after 72 h of *B. cinerea* infection. *BAM1* gene expression data were normalized to the NM controls. Three biological replicates were used in these analyses, and each replicate belonged to a different experiment involving a pool of leaf material from three individual plants per condition. The error bars represent the SE of the mean. Statistically significant differences are indicated in (A), (B), and (D) by different letters (ANOVA, Fisher’s least significant difference test; *P*<0.05, *n*=9); and in (C) by an asterisk (*t*-test; *n*=9).

Recently, our research group demonstrated that starch is a possible source of sugars for callose priming mediated by an increase in the β*-amylase 1* gene (*BAM1*) (Gamir *et al.*, 2018). The *BAM1* gene encodes a β-amylase responsible for the hydrolysis of starch into maltose in the chloroplast, which is transported to the cytoplasm and hydrolysed into glucose. Since sugar metabolism is among the main targets of AMF, it is plausible that the starch mobilization imposed by symbiosis (Gutjahr *et al.*, 2009) mediates the priming of callose deposition upon a shoot pathogenic infection. To study this hypothesis, we analysed the starch and ADP-glucose content and the *BAM1* gene expression level. The levels of ADP-glucose, which is the main precursor of starch biosynthesis, in the AM samples were augmented compared with those in the NM plants regardless of *B. cinerea* infection status (Fig. 2A). Notably, the starch content in the AM plants was higher than that in the NM plants (Fig. 2B). Interestingly, the infection induced starch mobilization, leading to a significant reduction in both the NM and AM plants, but the rate of starch degradation was higher in the AM plant by 15.75% (Fig. 2C). This stronger degradation correlated with significantly higher levels of *BAM1* gene expression in the AM plants compared with that in the NM plants, and the expression was further boosted upon pathogen infection (Fig. 2D).

**Fig. 3.**
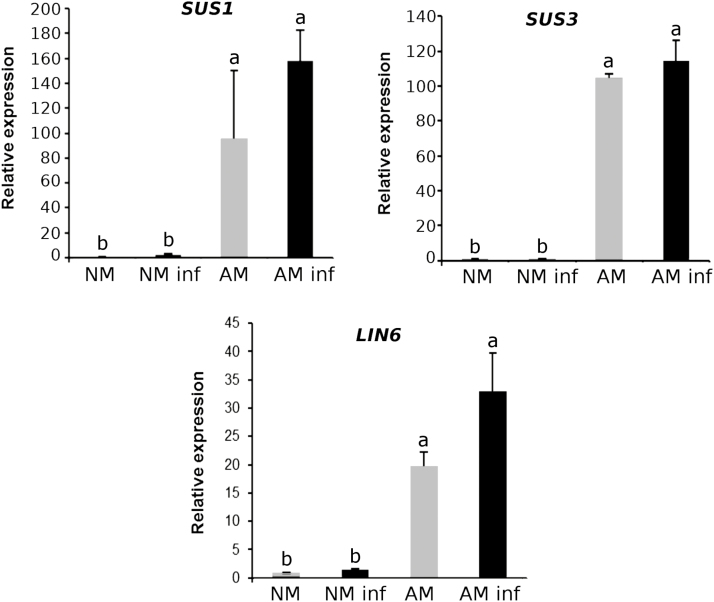
Expression levels of sucrose synthase (*SUS*) and a sugar invertase (*LIN6*) genes were up-regulated in mycorrhizal (AM) plants. Relative gene expression levels of the sucrose synthases *SUS1* and *SUS3* and the invertase *LIN6* in the non-mycorrhizal (NM) and AM tomato plants upon pathogenic infection (72 hpi, black bars) and control plants (grey bars). Three biological replicates of three different experiments were used in these analyses, and each replicate consisted of a pool of leaf material from three individual plants per condition. The SE of the mean is represented by the error bars, and statistically significant differences are indicated by different letters (ANOVA, Fisher’s least significant differencetest; *P*<0.05, *n*=9).

**Fig. 4.**
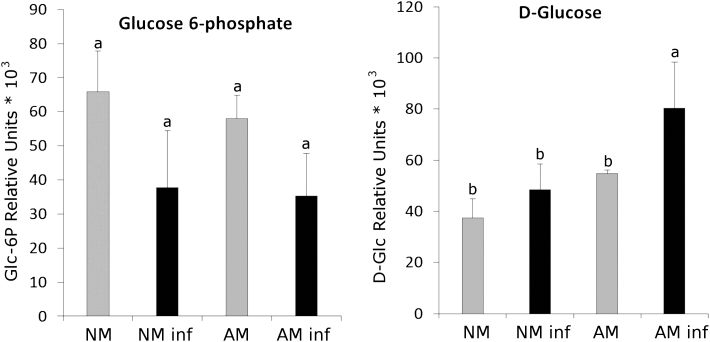
Sugar metabolism in mycorrhizal (AM) and non-mycorrhizal (NM) plants before and after *B. cinerea* infection. Levels of glucose 6-phosphate and d-glucose in the non-infected (grey bars) and infected (black bars) NM and AM tomato plants 72 h after *B. cinerea* infection. Leaf material from three different plants was pooled for each treatment combination. Each experiment was repeated three times. The error bars represent the SE of the mean. The letters indicate statistically significant differences (ANOVA, Fisher’s least significant difference test; *P*<0.05, *n*=9).

Many sucrose synthases and invertases showed higher levels in the AM plants (Schaarschmidt *et al.*, 2006; Li *et al.*, 2012). The most studied invertase in AM plants is LIN6, which is interestingly a JA-responsive tomato cell wall invertase. To decipher sugar metabolism during MIR and its possible link with primed callose accumulation, we also analysed the most studied sucrose synthase genes in tomato in response to either mycorrhization or pathogenic attack: *SUS1* (also known as *TOMSSF*), *SUS3*, and *LIN6* (García-Rodríguez *et al.*, 2007; Hyun *et al.*, 2011; Ruan, 2014). The *SUS1*, *SUS3*, and *LIN6* gene expression levels were higher in the AM plants in both the *B. cinerea*-infected and uninfected plants, whereas the NM plants showed a very low basal expression level of all three genes (the two synthases and the invertase) regardless of the infection status (Fig. 3). Sucrose synthases and invertases are known to be regulated not only at the transcriptional and post-transcriptional level but also at the post-translational level (reviewed by Wan *et al.*, 2018); hence, the study at the gene expression level performed in this work does not provide the complete picture of carbon metabolism. Nevertheless, previous studies support the role of these genes in symbiosis; for example, studies performed in *Medicago truncatula* showed that knocked down *MtSucS1* mutants were impaired in fungal colonization and caused an early collapse of arbuscules (Baier *et al.*, 2010). However, notably, ADP-glucose can be produced not only from ADP-glucose pyrophosphorylase (AGPase) activity but also from cytosolic sucrose synthase activity. Thus, the enhanced expression of the *SUS1* and *SUS3* genes could support the increase in the ADP-glucose content found in the AM plants regardless of infection with *B. cinerea* (Fig. 2A). Regarding sugar contents, in the absence of infection, the glucose-6-phosphate levels did not differ between the NM and AM plants, whereas a significant increase in glucose content was observed only in the AM plants upon pathogen infection, suggesting a higher availability to fuel callose accumulation (Fig. 4).

### Sugar transport in response to pathogens is altered in mycorrhizal plants

**Fig. 5.**
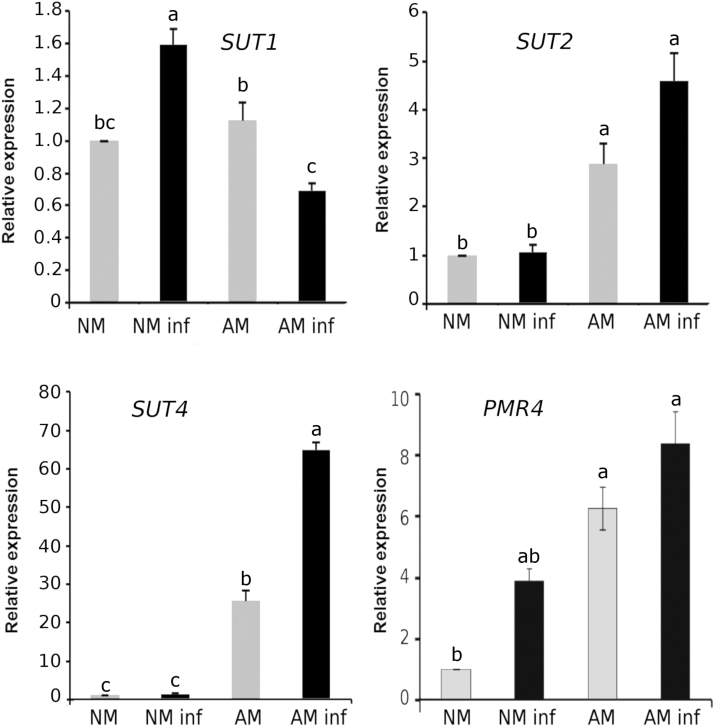
Sugar transport in response to *B. cinerea* infection in mycorrhizal (AM) and non-mycorrhizal (NM) plants and expression of the callose synthase *PMR4* gene. Expression levels of sugar transporter genes (*SUT1*, *SUT2*, and *SUT4*) and the *PMR4* gene in NM and AM infected (black bars) and non-infected (grey bars) tomato plants at 72 hpi. Three biological replicates were used in these analyses, and each replicate belonged to a different experiment involving a pool of leaf material from three individual plants per condition. The error bars represent the SE of the mean. and the statistically significant differences are indicated by letters (ANOVA, Fisher’s least significant difference test; *P*<0.05, *n*=9).

Carbohydrates, such as sucrose, required in organs other than leaves are loaded into the phloem for translocation to the sink organs. In tomato, three sucrose transporter genes have been identified: *SlSUT1*, *SlSUT2*, and *SlSUT4* (Osorio *et al.*, 2014). SlSUT1 and SlSUT2 are involved in phloem loading and unloading, respectively, whereas SlSUT4 is thought to be a vacuolar sucrose transporter (Endler *et al.*, 2006; Hackel *et al.*, 2006). The basal expression of the genes responsible for phloem unloading (*SUT2*) and vacuolar transport of sucrose (*SUT4*) in the AM plants was higher than that in the NM plants. Additionally, the expression of both genes was unaltered by the pathogen in the NM plants but was further boosted upon *B. cinerea* infection in the AM plants. Interestingly, *SUT1* (involved in phloem loading) had a completely different expression profile. *SUT1* was not induced by mycorrhiza but, in contrast, was induced in response to the pathogen in the NM plants, and the infected AM plants showed a strong reduction (Fig. 5). This observation suggests that sugars may be retained in the cytoplasm of AM plants following infection rather than being distributed to other metabolic sinks, including the roots. Recently, members of the sugar transporter *SWEET* family were shown to play an important role in mycorrhizal symbiosis and resistance to *B. cinerea* since they are involved in the release of sugars to the apoplast (Asai *et al.*, 2016; Manck-Götzenberger and Requena, 2016). In tomato plants, *SlSWEET15* has been described as hijacked by *B. cinerea* to obtain sucrose from the plant during the early stages of infection (Asai *et al.*, 2016). Under our experimental conditions, *SWEET15* did not change in the AM plants or in response to the infection (Supplementary Fig. S3). Nevertheless, the other members of the *SWEET* gene family presented lower (*SlSWEET17*) and higher levels (*SlSWEET4*) upon *B. cinerea* infection regardless of the mycorrhizal status of the plant (Supplementary Fig. S3), suggesting that both respond to the pathogen but are not relevant for MIR.

**Fig. 6.**
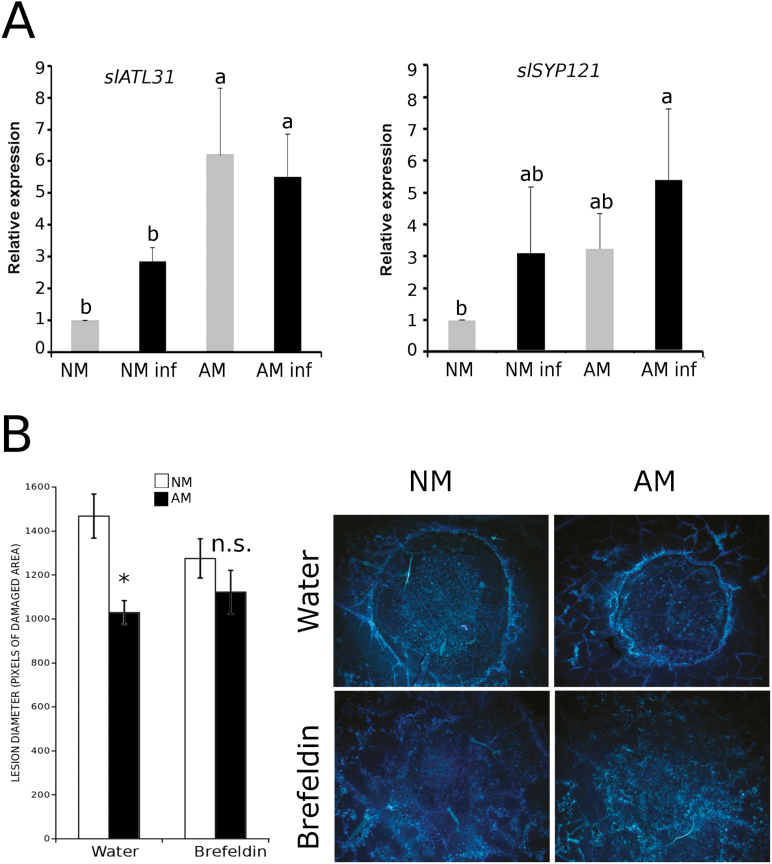
Vesicular trafficking role during MIR. (A) Expression level of the Q-SNARE *SYP121* gene and the ubiquitin ligase *ATL31* gene in non-infected (grey bars) or infected (black bars) non-mycorrhizal (NM) or mycorrhizal (AM) plants. The analysis was replicated three times in which three individual plants per treatment were pooled. The error bars represent the SE. The significant differences are indicated by letters (ANOVA, Fisher’s least significant difference test; *P*<0.05, *n*=9). (B) Lesion diameter caused by a drop in *B. cinerea* 72 hpi in NM (white bars) and AM (black bars) plants treated with brefeldin A to inhibit vesicular trafficking or with water as a control 24 h before infection. The error bars represent the SE of the mean, and (*) represents statistically significant differences (*t*-test; *n*=18). (This figure is available in colour at *JXB* online.)

The callose synthase PMR4 is usually regulated at the protein level, but transcriptional regulation has been observed in some specific situations (Ellinger and Voigt, 2014). Here, we observed the transcriptional regulation of callose synthase during symbiosis as the *PMR4* gene expression level was >6-fold higher in the AM plants independent of the pathogen infection (Fig. 5). In Arabidopsis, callose synthase has also been demonstrated to be spatially regulated by membrane trafficking by being transported via vesicular trafficking (Xie *et al.*, 2012; Ellinger *et al.*, 2013), and the final distribution of the PMR4 protein in the plasma membrane is driven by the Q-SNARE/R-SNARE complex (Meyer *et al.*, 2009). One of the Q-SNARE proteins, SYP121, has been shown to be involved in the Arabidopsis resistance against *Blumeria graminis* (Maekawa *et al.*, 2014). In addition, SYP121 interacts with the ubiquitin ligase ATL31, which is also important in fungal penetration. The ATLs are a large family of membrane-associated ubiquitin ligases (Guzmán, 2012). In Arabidopsis, two members of this family, namely ATL6 and ATL31, are specifically involved in callose accumulation during the plant immune response. Under our experimental conditions, the expression level of the *SlATL31* gene was enhanced in the AM plants compared with that in the NM plants, but the expression remained at similar levels upon pathogen infection. Nevertheless, *SlSYP121* gene expression was significantly enhanced only in the infected AM plants compared with the NM plants (Fig. 6A), suggesting a higher vesicular trafficking in AM plants in response to pathogen infection. To further investigate the role of vesicular trafficking in sugar transport to fuel callose priming during MIR, we treated the samples with BFA, which is an inhibitor of vesicular trafficking, 24 h before the leaf infection. The water-infiltrated leaves from the AM plants displayed MIR and callose priming. However, the BFA infiltration abolished normal callose accumulation in both the NM and AM plants, although some spots of callose were still observed in the AM plants (Fig. 6B). Remarkably, MIR was completely lost in the AM plants, supporting the key role of callose priming and vesicular trafficking in MIR.

### Proposed model of the priming of callose deposition during mycorrhiza-induced resistance

**Fig. 7.**
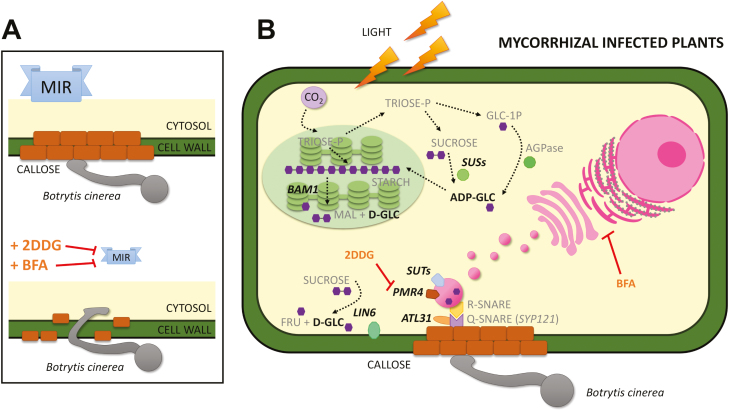
Proposed model of the priming of callose deposition in mycorrhizal tomato plants. (A) Role of callose in mycorrhizal-induced resistance (MIR). Callose deposition in the cell wall prevents fungal penetration during MIR (on top); however, the application of 2DDG (a PMR4 callose synthase inhibitor) or BFA (a vesicular trafficking inhibitor) blocks callose accumulation, allowing pathogen penetration. (B) Model depicting the proposed molecular mechanisms underlying the priming of callose deposition during MIR. 2DDG and BFA inhibitors are shown next to their targets. The words in bold show the enzymes and sugars that are accumulated in the mycorrhizal-infected plants compared with the non-mycorrhizal-infected plants. Monosaccharides and disaccharides are represented by hexagons. The dotted arrows could represent several metabolic steps. Abbreviations: 2-DDG, 2-deoxy-d-glucose; ADP-GLC, ADP-glucose; AGPase, ADP-glucose pyrophosphorylase; BAM1, ββamylase 1; BFA, brefeldin A; D-GLC, d-glucose; FRU, fructose; GLC-1P, glucose-1-phosphate; MAL, maltose; SUSs, sucrose synthases, SUT, sucrose transporter. (This figure is available in colour at *JXB* online).

Based on all the results presented above, we propose the model presented in Fig. 7. MIR triggers callose deposition in the cell wall, contributing to a reduction in fungal penetration (Fig. 7A, top); however, treatments with a PMR4 callose synthase inhibitor (2DDG) or a vesicular trafficking inhibitor (BFA) block callose accumulation, facilitating pathogen penetration (Fig. 7A, bottom).

A more detailed model illustrating the potential regulatory elements in the priming of callose deposition during MIR revealed in this work is presented in Fig. 7B. The higher gene expression and sugars levels in the infected AM plants compared with those in the infected NM plants are represented in bold. The gene expression of β*-amylase 1* in the infected AM plants is higher than that in the infected NM plants, improving the rate of starch degradation (Fig. 2). The sugar metabolism-related enzymes sucrose synthases, the invertase LIN6, and sucrose transporters are also more highly expressed in the AM infected plants (Figs 3, 5), which may lead to higher sugar mobilization that might contribute to higher callose deposition. ADP-glucose, which is the product of sucrose degradation by sucrose synthases, is increased in infected AM plants (Fig. 2A). d-Glucose, which is derived from either starch or sucrose degradation, also shows higher levels upon pathogen infection in these plants (Fig. 4). In addition, the infected AM plants displayed enhanced ubiquitin ligase *ATL31* gene expression (Fig. 6), strongly suggesting the importance of vesicular trafficking in callose accumulation during MIR.

These data are summarized in Fig. 7B, where the higher gene expression and sugar levels in the infected AM plants compared with those in the infected NM plants are represented in bold. The impairment of MIR by 2DDG and BFA and the targeted processes are also represented, supporting the key role of callose deposition priming in induced resistance.

## Discussion

Mycorrhiza-induced resistance against several biotic stressors, such as biotrophic and necrotrophic pathogens, has been investigated (Fiorilli *et al.*, 2011; Nair *et al.*, 2015; Sánchez-Bel et al., 2016). In this study, we found a more pronounced accumulation of callose in leaves of AM plants upon infection by the fungal pathogen *B. cinerea*. The inhibition of this primed callose response by previous infiltration with the callose synthase inhibitor 2DDG abolished the protection by AMF against *B. cinerea* confirming the relevance of callose priming in MIR (Fig. 1). Remarkably, simultaneous nitrogen depletion and infection by the necrotrophic fungus also abolish callose priming, explaining the partial MIR impairment observed under these conditions (Sánchez-Bel et al., 2016; Supplementary Fig. S1). These results strongly suggest that priming of callose deposition is critical for MIR against *B. cinerea*; however, in the absence of callose priming, MIR may be still functional. Notably, the priming of callose deposition has been reported as the main mechanism underlying the induced resistance achieved after chemical priming with compounds, such as I3CA and β-aminobutyric acid (BABA), against the necrotrophic pathogens *B. cinerea* and *P. cucumerina* (Nishimura *et al.*, 2003; Ton and Mauch-Mani, 2004; Gamir *et al.*, 2018). To the best of our knowledge, only one report investigated the role of callose deposition in MIR (Lee *et al.*, 2005). The authors observed that the priming of callose deposition seems to be the mechanism underlying the enhanced resistance against *Colletotrichum orbiculare* in mycorrhizal cucumber plants.

MIR against *B. cinerea* in tomato plants is mostly under the control of the JA signalling pathway (Sánchez-Bel et al., 2016). Here, we also observed the priming profile of JA (Supplementary Fig. S2), supporting its likely implication in enhanced callose accumulation. In addition, the JA-regulated invertase LIN6 showed a priming profile, and the expression of the vesicular sugar transporter *SUT4* gene was primed in the AM plants following infection (Figs 3, 5). Altogether, we collected evidence supporting the role of sugar mobilization and vesicular transport in callose accumulation, which may reconfigure the priming of the callose pathway in AM plants upon fungal infection.

It is commonly accepted that callose is accumulates at the plasma membrane by a glucan synthase complex which is thought to contain a GTPase, UDP-glucose transferase, and sucrose synthase, among other interactors (Ellinger and Voigt, 2014). According to this hypothesis, most aspects related to carbohydrate metabolism and transport might be important in the callose priming pathway. Recently, several studies investigated the mechanisms and molecular events controlling the callose synthesis and callose priming pathway (Maekawa *et al.*, 2014; Gamir *et al.*, 2018; Singh *et al.*, 2018). In the present study, the AM plants showed more active carbohydrate metabolism. ADP-glucose, which is the main precursor of starch biosynthesis, was accumulated more in AM plants than NM plants. Similarly, the starch levels in the AM plants were higher than those in the NM plants. Upon infection, a higher starch degradation rate is likely to be the most relevant event in AM plants contributing to callose priming (Fig. 2). Degradation may supply the sugars that need to be transported and polymerized to accumulate callose at the cell wall interface. To test this hypothesis, the sugar metabolism and expression of the most relevant sugar transport and synthesis genes were analysed.

Host sugar reallocation is an important event during AM symbiosis. AMF take up hexoses mostly as glucose derived from sucrose degradation by the invertases and sucrose synthases in the host cell (Pfeffer *et al.*, 1999; Bago *et al.*, 2003). AMF achieve this presumably via mechanisms similar to those by which pathogenic fungi hijack the sucrose degradation and transport machinery of the host cell (Proels and Hückelhoven, 2014). Therefore, some invertases and their hexose products have been suggested to play a key regulatory role linking the plant sugar signalling pathways and AM symbiosis (Schaarschmidt *et al.*, 2007).

In this study, the expression levels of the genes encoding the sucrose synthases SUS1 and SUS3 and the invertase LIN6 were higher in the AM plants (Fig. 3). LIN6 was previously shown to be induced in shoots and roots of AM tomato plants and is thought to be responsible for the hydrolysis of the sucrose delivered to the shared apoplast between the AMF and the plant (García-Rodríguez *et al.*, 2007). Under our experimental conditions, we did not observe any *LIN6* induction in the NM infected plants, while this induction was significantly higher in the AM plants upon *B. cinerea* infection. This result suggests that the higher glucose content found in the *B. cinerea*-infected AM plants (Fig. 4) may result from both the different carbohydrate metabolism between NM and AM plants (higher starch hydrolysis and sucrose degradation) and the lower consumption of glucose by *B. cinerea* due to a lower infection rate. Hexose pools and the sucrose:trehalose-6-phosphate ratio are emerging as important events in cell signalling determining cellular responses (Bolouri Moghaddam and Van den Ende, 2012; Lunn *et al.*, 2014; Figueroa and Lunn, 2016). Although glucose, sucrose, and trehalose-6-phosphate are the sugars most studied as signalling metabolites in plants, UDP-glucose (UDP-Glc) is emerging as an intracellular mediator of reactive oxygen species (ROS) signalling and plant cell death in plants (Janse van Rensburg and Van den Ende, 2017). Considering that UDP-Glc is the main substrate of callose synthase, it is reasonable to hypothesize that under our experimental conditions, the UDP-Glc levels in the AM and NM plants could be different and may play a role in stress signalling. Notably, we found an enhanced callose accumulation rate in the *B. cinerea*-infected AM plants compared with that in the NM infected plants, which could indicate higher PMR4 activity upon *B. cinerea* infection and, therefore, a greater use of UDP-Glc in these plants.

Consistent with this hypothesis, the elevated *PMR4* gene expression level found in the AM plants did not correlate with any callose accumulation in the uninfected leaves (Figs 1, 5), suggesting that post-transcriptional and/or post-translational regulation of PMR4, as well as the spatial regulation by vesicular trafficking, may define the final output of callose accumulation. In Arabidopsis, PMR4 is transported to the plasma membrane through intracellular vesicles (Xie *et al.*, 2012; Ellinger *et al.*, 2013) and redistributed to the penetration sites during fungal infection (Meyer *et al.*, 2009). The fusion of the tethered vesicles to the plasma membrane is mediated by specific binding between donor membrane-associated proteins, known as R-SNAREs, and plasma membrane-associated proteins, known as Q-SNAREs (Gu *et al.*, 2017). In tomato, an orthologue of the vesicular trafficking-associated *ATL31* has been characterized, suggesting that both Arabidopsis and tomato share the same mechanism (Lu *et al.*, 2016). In the experiments presented in this work, *SlATL31* and *SlSYP121* (a Q-SNARE) expression was induced in the AM plants. Furthermore, *SlSYP121* expression was further boosted upon *B. cinerea* infection, suggesting that both genes contribute to callose deposition during MIR. In fact, the inhibition of membrane trafficking by the BFA treatments completely abolished callose deposition and consequently MIR, confirming that functional vesicular trafficking is needed for callose priming during MIR (Fig. 6).

The transport and sugar level imbalances suggest that AM plants upon infection may orchestrate starch degradation and sugar transport into vesicles, mobilizing sugars within the cell through *BAM1*, *LIN6*, and *SUT4*. Thus, AM plants have a higher availability of carbohydrates probably due to both a higher rate of photosynthesis (Sánchez-Bel *et al.*, 2018) and more active starch degradation metabolism. At the end of the cellular pathway leading to callose deposition, membrane trafficking (estimated through *SlATL31* and *SlSYP121* gene expression levels) is also enhanced in AM-infected plants. Finally, *PMR4* gene expression was also higher in the AM plants with the AMF–host plant interaction being one of the specific cases in which *PMR4* transcriptional regulation is found. Notably, the higher basal levels of *PMR4* could allow faster activation of the response by post-transcriptional modifications (Fig. 7). Interestingly, our results reveal an important overlap between the priming of the callose pathway described in Arabidopsis (Gamir *et al.*, 2018) and that occurring in AM tomato plants, suggesting that callose priming is a conserved mechanism underlying defence priming triggered by different priming stimuli in different plant species.

## Supplementary data

Supplementary data are available at *JXB* online.

Table S1. List of primers used for qPCR analysis.

Fig. S1. Callose deposition under nitrogen starvation and normal fertilization in infected non-mycorrhizal and mycorrhizal tomato plants.

Fig. S2. Targeted analysis of oxylipin hormones levels in non-mycorrhizal and mycorrhizal plants at 72 hpi.

Fig. S3. Expression levels of different *SWEET* sugar transporter genes during MIR.

eraa030_suppl_Supplementary_Table_S1_Figure_S1_S3
