# Supplementary material for: Role and mechanisms of callose priming in mycorrhiza-induced resistance
Source: J Exp Bot. 2020 Jan 27;71(9):2769–81. doi: 10.1093/jxb/eraa030 (PMC7210776; doi:10.1093/jxb/eraa030)
Supplement: eraa030_suppl_Supplementary_Table_S1_Figure_S1_S3 [file eraa030_suppl_supplementary_table_s1_figure_s1_s3.pdf]

Supplementary Table 1. List of primers used for qPCR analysis.

Primer name	PCR primers sequence (5’ → 3’)	Gene ID (NCBI)
ACT-52	F – CACCATTGGGTCTGAGCG<br>R – GGGCGACAACCTTGATCT	101263261
ATL31	F – CGTACTGCTGCTGCTACTCG<br>R – CATGAGATTTGAGCCAAGCA	101265719
BAM1	F – TCGAGTTCTGTTATGCGTTTTG<br>R – TCTCCTCAACAACCCACCTC	100736451
BC-TUB	F – CCGTCATGTCCGGTGTTACCAC<br>R – CGACCGTTACGGAAATCGGAA	5441652
EF-1	F – GATTGGTGGTATTGGAACTGTC<br>R – AGCTTCGTGGTGCATCTC	101244084
LIN6	F – AGCACATTTATTCGCCTTCAAC<br>R – TTTGTGACGTGGCATAATAAGA	543502
PMR4	F – GCCGGCGGCGAGACAAGTTT<br>R – CAGCGCCAGCCAGTCAAGCA	101243903
SUS1	F – GGATTGAAAGCCACGGAAAGG<br>R – ACCAGGCCTCAACGAATAGCA	543961
SUS3	F – GGTTTCTGTCTGATTGTTATCC<br>R – ACAGAAGGGAAAAATGGCAAA	543731
SUT1	F – TTCCATAGCTGCTGGTGTTC<br>R – TACCAGAAATGGGTCCACAA	544099
SUT2	F – CCTACAGCGTCCCTTTCTCT<br>R – GGATACAACCATCTGAGGTACAA	543613
SUT4	F – TCTCCGCTGATATTGGATGG<br>R – GCAACATCGAGAAGCCAAAA	543620
SWEET4	F – CTGGGACGATGAAGAAAAGC<br>R – GTGGCTTTAAACTGTGTCCG	101246848
SWEET15	F – ACGCAAAGCCAGCGATAGAT<br>R – ACGTCTTCGGAATCAACAGG	101255592
SWEET17	F – GCTCGGCCCATCTTATGAAA<br>R – GGCAGGCTTTTCCATTTGTG	101255592
SYP121	F – AAGGCAAAGAATGGCATCTG<br>R – TGTCCTCTTCCTTGCTCCTG	101247381

Supplementary Figure 1

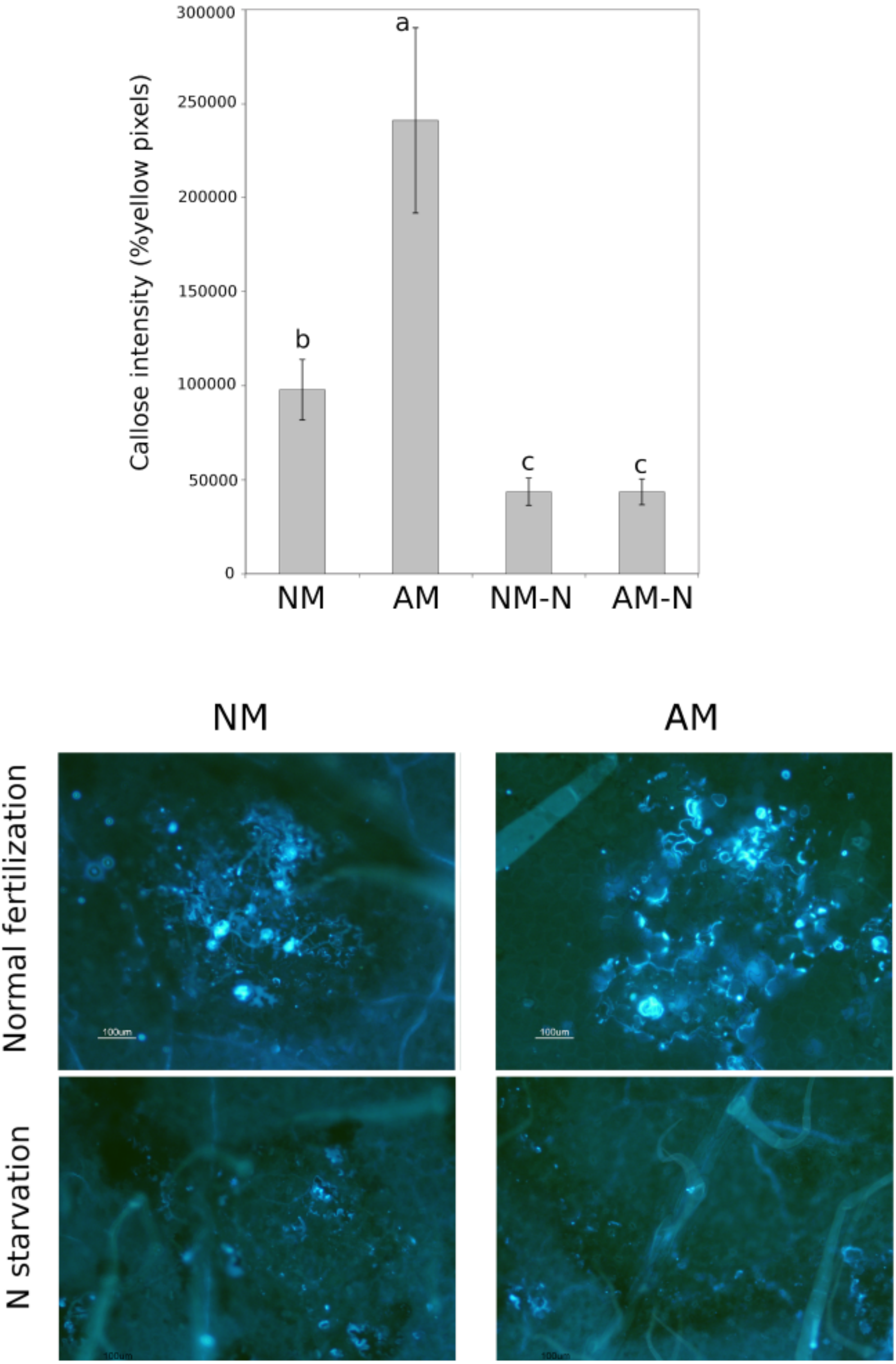

Supplementary Figure 1. Callose deposition upon *Botrytis cinerea* infection under Nitrogen starvation. (A) Level of callose in non-mycorrhizal (NM) and mycorrhizal (AM) infected tomato plants under normal fertilization and under 48h of nitrogen starvation (–N). Three different replicates, which consist in three pooled plants per condition, were carried out. Standard error is represented by error bars. (*) represents statistically significant differences (T-test; n = 9).

Supplementary Figure 2

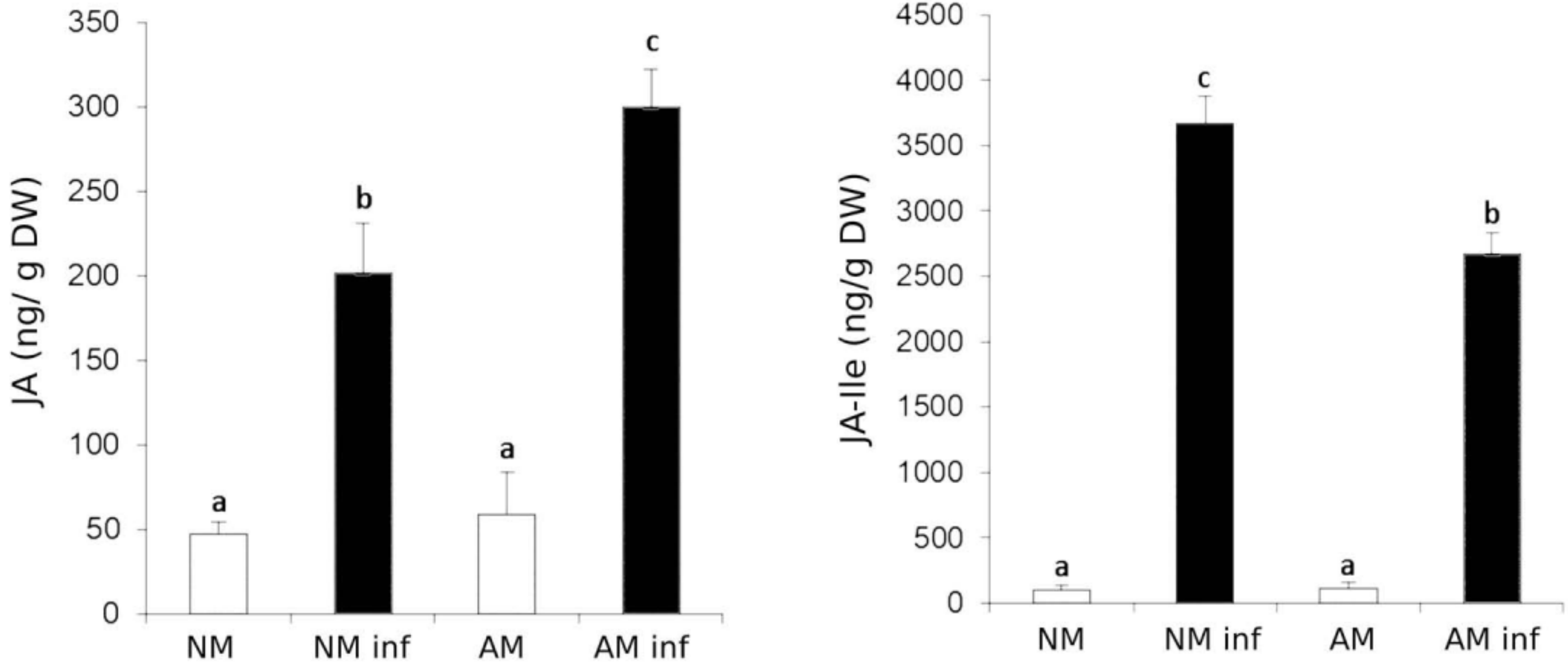

Supplementary Figure 2. **Oxylipin levels.** Targeted analysis of oxylipin hormones, JA and JA-isoleucine, in non-mycorrhizal (NM) and mycorrhizal (AM) plants 72 hpi. For each combination, infected leaves from three plants were pooled. Experiments were repeated three times to have different replicates. Letters indicate statistically significant differences (ANOVA, Fisher's least significant difference [LSD] test; $p < 0.05$, $n = 9$).

Supplementary Figure 3.

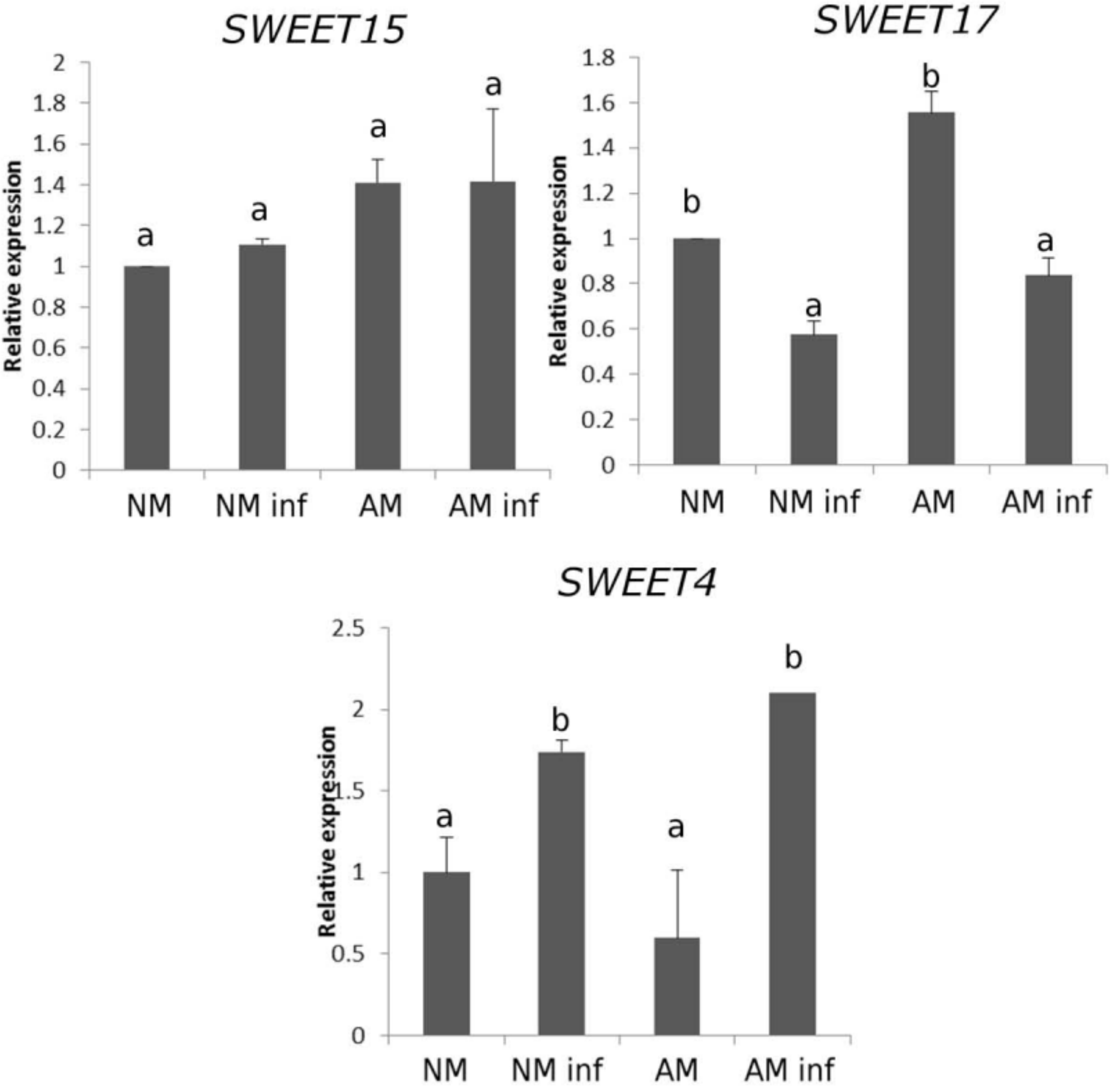

Supplementary Figure 3. **Expression levels of different *SWEET* sugar transporter genes**. Levels of *SWEET* sugar transporters (4, 15 and 17) gene expression in non-mycorrhizal (NM) and mycorrhizal (AM) non-infected and infected (inf) tomato plants at 72 hpi. Experiments were repeated three times to have different replicates, which consist in three pooled plants for each combination. The standard error of the mean is represented by error bars and statistically significant differences are represented by different letters (ANOVA, Fisher's least significant difference [LSD] test; $p < 0.05$, $n = 9$).
